# Continuous multistep synthesis of 2-(azidomethyl)oxazoles

**DOI:** 10.3762/bjoc.14.36

**Published:** 2018-02-23

**Authors:** Thaís A Rossa, Nícolas S Suveges, Marcus M Sá, David Cantillo, C Oliver Kappe

**Affiliations:** 1 Institute of Chemistry, University of Graz, NAWI Graz, Heinrichstrasse 28, 8010 Graz, Austria; 2 Departamento de Quıímica, Universidade Federal de Santa Catarina, Florianópolis 88040-900, SC, Brazil; 3 Chemistry Institute, Federal University of Rio de Janeiro, Rio de Janeiro, RJ, Brazil 22941-909,; 4 Research Center Pharmaceutical Engineering GmbH (RCPE), Inffeldgasse 13, 8010 Graz, Austria

**Keywords:** azirines, continuous flow, heterocycles, oxazoles, process integration, vinyl azides

## Abstract

An efficient three-step protocol was developed to produce 2-(azidomethyl)oxazoles from vinyl azides in a continuous-flow process. The general synthetic strategy involves a thermolysis of vinyl azides to generate azirines, which react with bromoacetyl bromide to provide 2-(bromomethyl)oxazoles. The latter compounds are versatile building blocks for nucleophilic displacement reactions as demonstrated by their subsequent treatment with NaN_3_ in aqueous medium to give azido oxazoles in good selectivity. Process integration enabled the synthesis of this useful moiety in short overall residence times (7 to 9 min) and in good overall yields.

## Introduction

**Figure 1:**
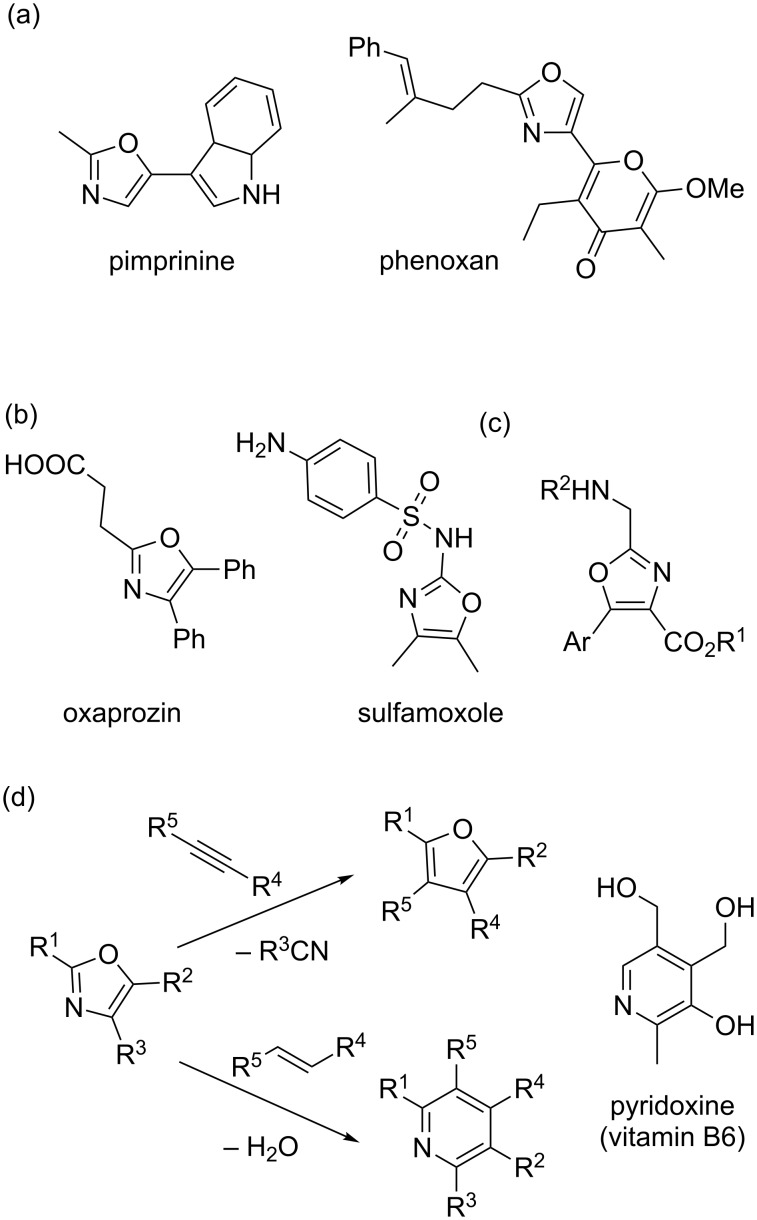
Examples of naturally occurring oxazoles (a); some drugs containing oxazole as the active moiety (b); general structure of fluorescent dipeptidomimetics derived from trisubstituted oxazoles (c); reactivity of the oxazole system as an azadiene (d).

Oxazoles are an important class of five-membered aromatic heterocycles containing one oxygen and one nitrogen atom in their structures. The oxazole moiety is relatively stable and is found widely in nature [1–3]. Naturally occurring oxazoles include compounds with antibiotic or antimicrobial properties such as pimprinine [4] or phenoxan [5] (Figure 1a). Also many synthetic active pharmaceutical ingredients (API) contain the oxazole as an active moiety [1–3]. Oxaprozin, for example, is an important non-narcotic, non-steroidal anti-inflammatory drug [6–7]. Sulfamoxole is a broad-spectrum antibiotic for the treatment of bacterial infections (Figure 1b) [8]. In addition, ongoing studies show the potential of amino and amido-oxazoles to act as fluorescent dipeptidomimetics (Figure 1c) [9]. Due to their diene character, oxazoles find also use as intermediates in the synthesis of other organic scaffolds such as furans and pyridines, via cycloaddition/retro-cycloaddition tandem processes (Figure 1d) [10–13]. A classical example is the preparation of pyridoxine (a form of vitamin B6) using this approach [14–15].

**Scheme 1:**
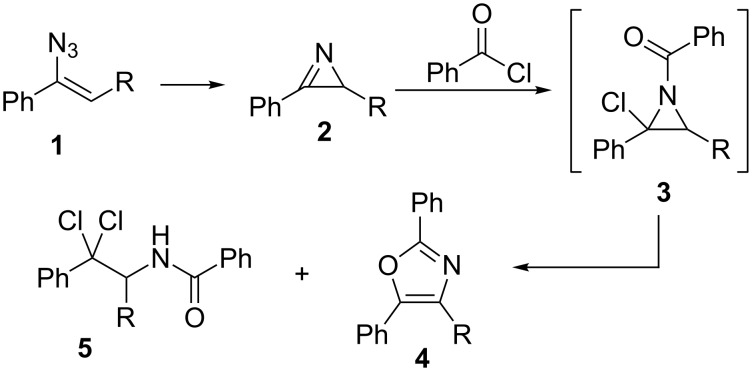
Synthesis of oxazoles **4** by addition of acyl chlorides to azirines **2**, as described by Hassner et al. [28–29].

There are several methods for the preparation of oxazoles described in the literature. These include ring-closure reactions of diazocarbonyl compounds with amides or nitriles [16], α-haloketones and amides [17–19], cyanohydrins and aldehydes (Fischer synthesis) [20–21], or oxidative additions of α-methylene ketones to nitriles [22–23]. An alternative approach consists of the ring expansion of azirines, which can be prepared from vinyl azides **1**, by the reaction with carbonyl compounds. Substituted 2-acylazirines rearrange to oxazoles in the presence of bases [24–26]. In addition the light-mediated synthesis of oxazoles from azirines and aldehydes also has been described by Lu and Xiao [27]. Hassner and Fowler described the reaction of azirines **2** with acyl chlorides with formation of intermediate adduct **3** to give oxazoles **4** in polar solvents (Scheme 1) [28–29]. In the latter reaction amide **5** was formed as a side-product and the aziridine intermediate **3** was stable and could be isolated when the reaction was carried out in non-polar solvents.

**Scheme 2:**
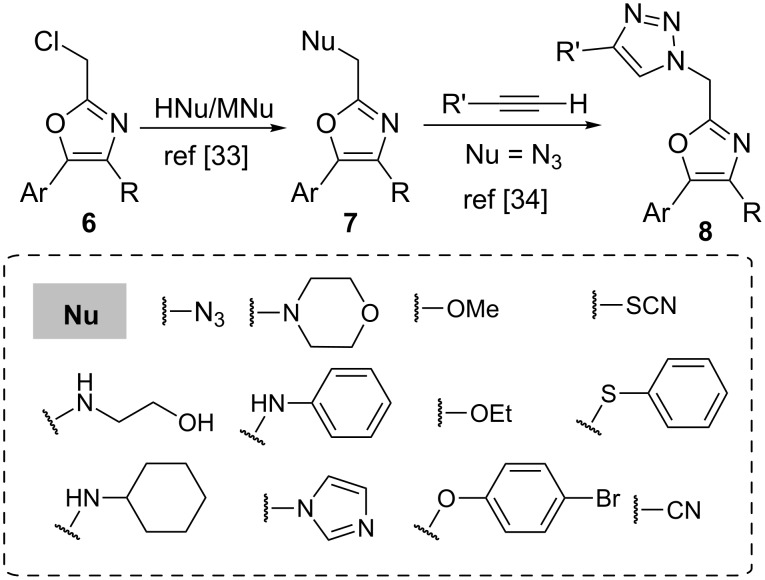
Preparation of 2-functionalized oxazoles **7** from 2-(chloromethyl)oxazoles **6** and their application to the synthesis of peptidomimetics **8**.

In particular, 2-(halomethyl)oxazoles **6** are a class of compounds rather underexplored, even though they are frequently key intermediates in the total synthesis of natural products [30–32]. Recently, Patil and Luzzio reported the preparation of a wide range of 2-substituted derivatives **7** by a simple nucleophilic halide displacement from 2-chloromethyl-4,5-diaryloxazoles, illustrating their usefulness (Scheme 2) [33]. In a related work, Luzzio et al. described the synthesis of 1,4-disubstituted triazoles **8** through click reaction between 2-azidomethyl-4,5-diaryloxazoles and alkynes in the presence of a copper(I) catalyst (Scheme 2). The authors were able to synthesize an array of small-molecule peptidomimetics that inhibited *Porphyromonas gingivalis* biofilm formation [34].

Important drawbacks observed in the generation of compounds of type **7** include the instability of the halide intermediate **6**, which might be difficult to isolate due decomposition reactions, as well as selectivity issues during the generation of the oxazole ring. It has been shown that problems associated with unstable intermediates or reagents can be overcome with the use of continuous-flow chemistry. Continuous-flow processing has demonstrated to be an ideal tool for the development of uninterrupted multistep reactions [35–37]. The integration of several sequential steps can be readily achieved through a continuous addition of reagent streams, quenching, liquid–liquid extraction, or even filtration stages, thus avoiding the handling of unstable intermediates [35–37].

**Scheme 3:**
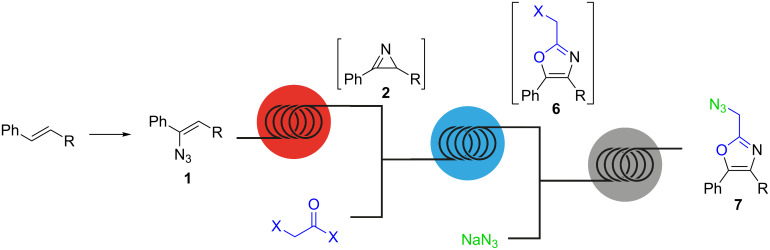
Integrated continuous-flow synthesis of 2-(azidomethyl)oxazoles **7**.

In this article we present an integrated continuous-flow procedure for the preparation of 2-(azidomethyl)oxazoles **7** starting from vinyl azides through an azirine intermediate (Scheme 3). The process starts with the generation of the azirine from the vinyl azide by thermolysis. Formation of azirines from vinyl azides by photolysis and thermolysis in continuous flow has been previously described [38–39]. The azirine intermediate is then reacted with a 2-haloacyl halide at room temperature, to form the 2-(halomethyl)oxazole moiety. Subsequent reaction with an aqueous stream containing NaN_3_ then leads to the formation of the desired 2-(azidomethyl)oxazole. The optimization of each reaction step and the integration to a fully continuous process are described in detail.

## Results and Discussion

### Thermolysis of the vinyl azide and oxazole formation. Batch optimization

The reaction conditions for the thermolysis of the vinyl azide and the subsequent ring expansion of the intermediate azirine to form the oxazole ring were initially optimized in batch. For these experiments, vinyl azide **1a** was used as a model substrate. The small-scale batch thermolyses were carried out using sealed 1.5 mL vials heated in an aluminum platform. A 0.5 M solution of substrate **1a** was prepared using acetone as the solvent. The experiments were carried out placing 0.5 mL of the solution in the vial, which was sealed with a crimp-cap. The reactions were performed at three different temperatures (130–150 °C, Table 1). Notably, at 150 °C a very fast (1 min) and clean reaction (>99% purity by HPLC–UV analysis) was achieved.

**Table 1:** Batch optimization of the thermolysis of vinyl azide **1a.**^a^

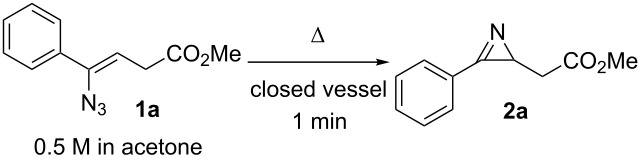			

entry	temp. (°C)	conv. (%)^b^	purity (%)^b^

1	130	88	>99
2	140	97	>99
3	150	>99	>99

^a^Conditions: **1a** in acetone (0.5 M), 0.5 mL solution in a 1.5 mL vial. ^b^Determined by HPLC peak area integration at 254 nm.

Next, the formation of 2-(bromomethyl)oxazole **6a** from azirine **2a** was also optimized under batch conditions. All reactions were carried out under an argon atmosphere using a 0.5 M solution of the substrate **2a** in acetone. In general, the addition of the reagents was performed at 0 °C in an ice-bath followed by stirring the reaction mixture at room temperature. When triethylamine (TEA) or *N,N*-diisopropylethylamine (DIPEA) were used as the base a solid formed after a few minutes in the reaction mixture (Table 2, entries 1–4), probably their corresponding ammonium bromide salts. Yet, good purities were achieved employing both bases and the incomplete conversions were ascribed to the presence of water in the reaction mixture. For this reason, further transformations were carried out in acetone dried over molecular sieves (3 Å). Using 1,5-diazabicyclo[4.3.0]non-5-ene (DBN) as the base (Table 2, entry 5) full conversion of the azirine **2a** was observed after 3 min reaction time. In this case the oxazole **6a** was formed with 74% purity and no formation of solids was observed. At longer reaction time (30 min) a slight decrease in purity could be detected, probably due to slow decomposition of **6a** (Table 2, entry 6). The reaction with chloroacetyl chloride instead of the bromo derivative delivered the corresponding (chloromethyl)oxazole with similar selectivity, the reaction was slower though, with 92% conversion being achieved after 10 min (Table 2, entry 7). The adjustment of other parameters such as the order of added reactants or variation in temperature showed little influence on the outcome of the reaction (Table 2, entries 8–10). Notably, oxazole **6a** was also formed in the absence of base (Table 2, entries 11–13). This was probably due to the weak basic character of the oxazole moiety itself, producing the oxazole hydrobromide. Finally, the variation of the amount of bromoacetyl bromide had no significant effect on the outcome of the reaction (Table 2, entries 12 and 13).

**Table 2:** Optimization of the reaction conditions for the generation of oxazole **6a** from azirine **2a**.^a^

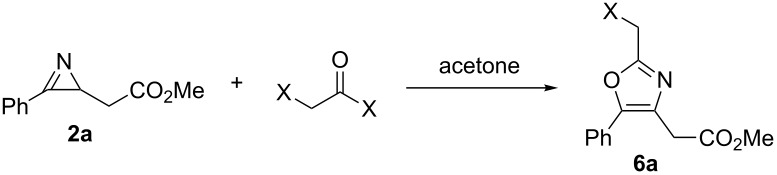							

entry	halide equiv	X	base (equiv)	time (min)	temp.	conv. (%)^b^	purity (%)^b^

1^c^	1.1	Br	TEA (1.1)	3	0 °C to rt	90	82
2^c^	1.1	Br	TEA (1.1)	30	0 °C to rt	94	77
3^c^	1.2	Br	DIPEA (1.1)	3	0 °C to rt	66	76
4^c^	1.2	Br	DIPEA (1.1)	30	0 °C to rt	76	77
5	1.1	Br	DBN (1.1)	3	0 °C to rt	>99	74
6	1.1	Br	DBN (1.1)	30	0 °C to rt	>99	70
7	1.1	Cl	DBN (1.1)	10	0 °C to rt	92	79
8^d^	1.1	Br	DBN (1.1)	5	0 °C to rt	>99	75
9^d^	1.1	Br	DBN (1.1)	5	−10 °C	>99	73
10^e^	1.1	Br	DBN (1.1)	4	rt	>99	81
11	1.1	Br	–	1	rt	>99	77
12	1.0	Br	–	1	rt	>99	79
13	1.3	Br	–	1	rt	>99	80

^a^Conditions: 0.50 mL solution of **2a** in acetone (0.5 M) was mixed with 0.50–0.65 mL of a solution of the acyl halide in acetone (0.5 M), followed by addition of the base (0.275 mmol). ^b^Determined by HPLC (254 nm) peak area integration. ^c^Formation of insoluble solid during the reaction. ^d^Base added after 3 min of reaction. ^e^Substrate added after 1 min of reaction.

**Scheme 4:**
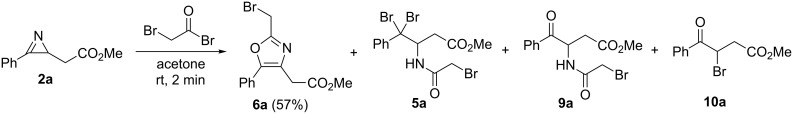
Side products generated during the reaction of azirine **2a** with bromoacyl bromide at room temperature.

To identify the reaction byproducts formed during the coupling of azirine **2a** and bromoacetyl bromide, the reaction was performed on a 3 mmol scale. The reaction mixture was quenched with NaHCO_3_ solution, extracted with ethyl acetate and concentrated under reduced pressure. The ^1^H NMR analysis of the crude product mixture revealed three major side products. Purification by column chromatography permitted the separation and isolation of each component (Scheme 4), which were characterized by ^1^H NMR, ^13^C NMR and low-resolution mass spectroscopy. In agreement with the observations by Hassner et al. [28–29], dibromoamide **5a** and its hydrolysis product **9a** were obtained in addition to ketoester **10a**. The distribution profile calculated by ^1^H NMR peak integration revealed a composition of 76% product **6a**, and side products in 7% (**5a**), 10% (**9a**) and 7% (**10a**), respectively. The oxazole **6a** was isolated as yellow solid (mp 76.3–78.1 °C) in 57% yield.

**Figure 2:**
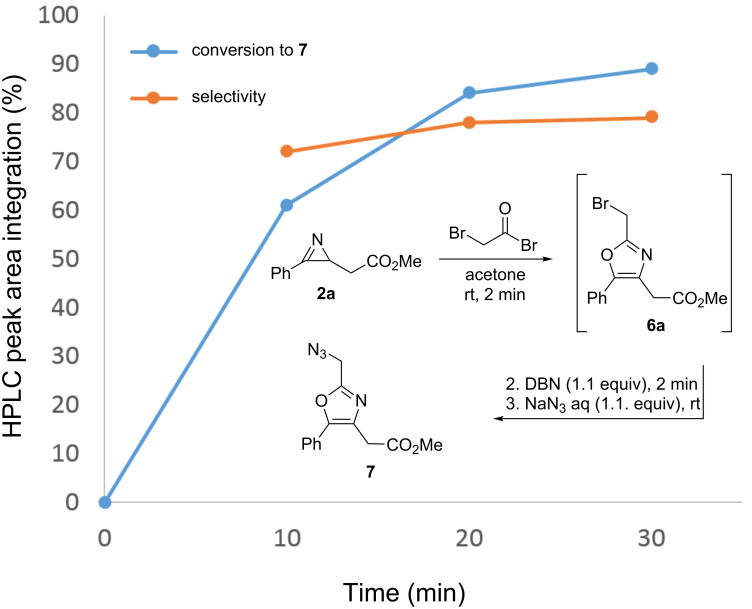
HPLC monitoring of the formation of 2-(azidomethyl)oxazole **7a**.

The optimization of the reaction conditions for the nucleophilic halide displacement with sodium azide were also evaluated in batch. A one-pot procedure starting from the azirine (without isolation of the 2-(bromomethyl)oxazole) was utilized to simulate the conditions of an integrated process. Thus, azirine **2a** was reacted with bromoacetyl bromide in a 1.5 mL vial using the conditions stated in Figure 2 (1.1 equiv bromide added at 0 °C, cf. Table 2, entry 5) in dry acetone for 3 min. Then, DBN was added to neutralize the acidic medium, followed by a 2.5 M aqueous solution of NaN_3_ (1.1 equiv) and the resulting mixture was stirred at room temperature. A conversion of 89% to **7a** from bromo oxazole **6a** with a selectivity of 74% was achieved after 30 min (Figure 2).

Subsequently, the amount of NaN_3_ was increased to 1.3 equiv to enhance the reaction rate. During the formation of the 2-(bromomethyl)oxazoles **6** addition of a base is not required (see Table 2, entries 11–13). However, neutralization of the resulting oxazole hydrobromide is required prior to the addition of NaN_3_ in order to avoid the generation of hydrazoic acid. Taking this into account, we decided to replace DBN by less expensive DIPEA for the subsequent reactions. Using both substrates **2a** and **2b**, it was observed that after 5 min of reaction at rt, the conversion of bromo oxazoles **6** into azido oxazoles **7** was up to 92% (Table 3, entries 1 and 4). When the reaction temperature was increased from rt to 50 °C, good conversions were achieved after 5 min reaction for both for the model substrate **2a** and the azirine **2b** (Table 3, entries 2 and 5). NaN_3_ was not fully soluble in the reaction mixture (after mixing with the acetone medium), which would be problematic for the later translation to flow conditions. Diluting the NaN_3_ solution from 2.5 M to 1.5 M (and therefore adding a larger volume of the solution to obtain the same excess of the reagent) resulted in fully homogeneous conditions suitable for flow processing (Table 3, entry 3).

**Table 3:** Batch optimization of the generation of 2-(azidomethyl)oxazoles **7a** and **7b**.^a^

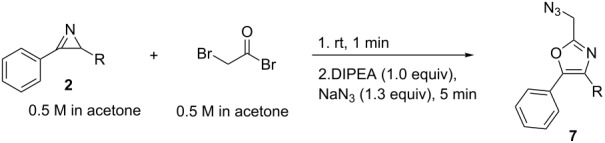					

entry	R	NaN_3_ conc. (M)	temp. (°C)^b^	conv. (%)^c,d^	purity (%)^d^

1	CH_2_CO_2_Me (**2a**)	2.5	rt	84	80
2	CH_2_CO_2_Me (**2a**)	2.5	50 °C	95	69
3	CH_2_CO_2_Me (**2a**)	1.5	50 °C	97	74
4	H (**2b**)	2.5	rt	92	69
5	H (**2b**)	2.5	50 °C	97	67

^a^Conditions: 0.4 mL of a 0.5 M solution of azirine in acetone, bromoacetyl bromide injected as a 0.5 M solution. ^b^Temperature for the reaction with NaN_3_. ^c^Conversion for the nucleophilic displacement step. ^d^Determined by HPLC (254 nm) peak area integration.

### Continuous-flow experiments

**Azirine formation.** With the optimal conditions for the three reaction steps in hand, we translated the process to continuous-flow conditions. For that purpose, individual continuous-flow reactors for each step were setup, the reaction conditions re-optimized when necessary, and finally all the steps integrated in a single continuous stream.

The thermolysis of vinyl azide **1** was performed in a continuous flow reactor consisting of a perfluoroalkoxy (PFA) coil (0.5 mL, 0.8 mm i.d.) immersed in a silicon bath at 150 °C. The vinyl azide solution in acetone was introduced into the reactor by a syringe pump (Syrris) with variable flow rates (Table 4) to obtain different residence times. The system was pressurized using a back-pressure regulator (BPR, Upchurch) at 17 bar (250 psi). The reaction mixture was cooled by immersing a second section of the coil reactor in an ice bath, to avoid damage of the BPR by the hot reaction mixture and evaporation of the solvent after the pressure release. Notably, flow rates had to be reduced with respect to those calculated for a residence time of 1 min for the generation of azirine **2a**, probably due to an expansion of the reaction mixture from the N_2_ generation, which reduced the actual residence time within the coil (Table 4, entries 1 and 2). Using the same flow setup azirines **2b** and **2c** were also successfully generated from the corresponding vinyl azides (Table 4, entries 3–5).

**Table 4:** Continuous-flow generation of azirines **2** by thermolysis of vinyl azides **1**.^a^

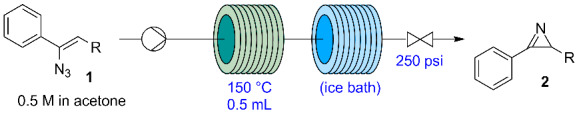					

entry	R	flow rate (µL/min)^b^	time (min)	conv. (%)^c^	purity (%)^c^

1	CH_2_CO_2_Me (**2a**)	500	1	98	100
2	CH_2_CO_2_Me (**2a**)	250	2	100	100
3	H (**2b**)	250	2	91	92
4	H (**2b**)	167	3	95	94
5	CH_2_OH (**2c**)	500	1	100	94

^a^Conditions: 0.5 M substrate in acetone, 5 mL reaction mixture (2.5 mmol) collected from the reactor output. ^b^Theoretical residence time calculated from the flow rate and reactor volume. ^c^Determined by HPLC (254 nm) peak area integration.

**Figure 3:**
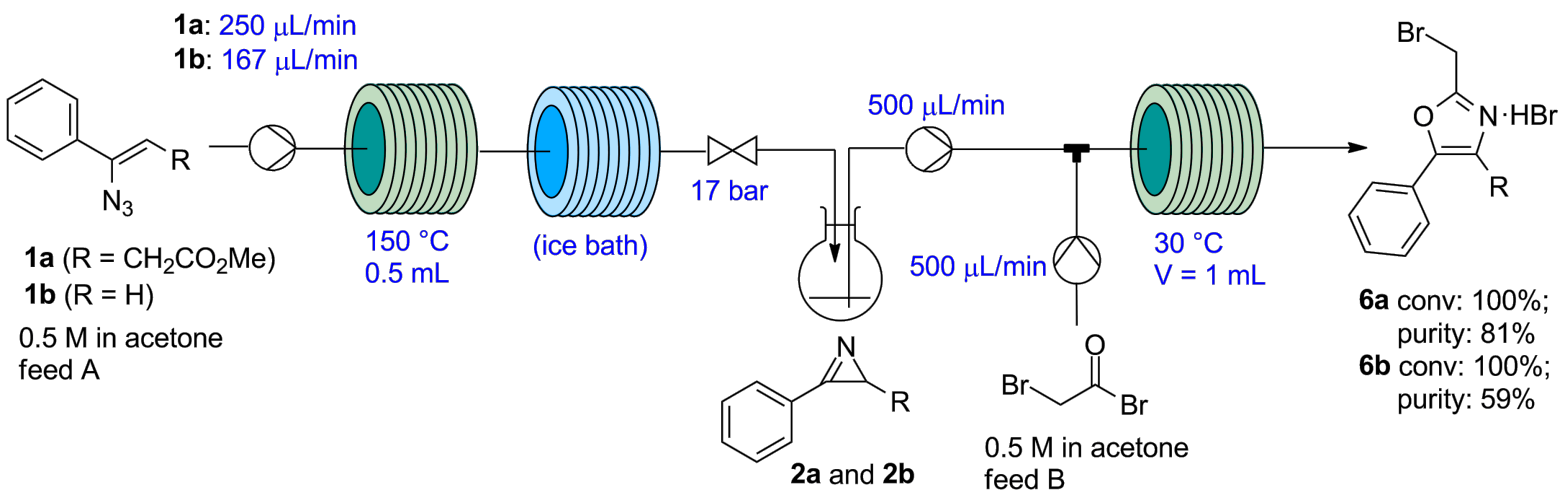
Continuous sequential thermolysis of vinyl azides **1** and ring expansion of azirines **2** with bromoacetyl bromide to give 2-(bromomethyl)oxazoles **6**.

The continuous-flow setup was then extended by incorporating a second reagent feed with a stream containing the bromoacetyl bromide solution (0.5 M in acetone, Figure 3). A vessel was placed between the two reaction zones to release the N_2_ generated during the azirine formation, which was maintained under argon atmosphere. Using this system, the crude reaction mixture obtained from the first reaction zone, containing the azirine in acetone, was directly pumped into the second reaction zone (500 µL/min), mixed with the bromoacetyl bromide stream in a T-mixer, and reacted at 30 °C in a PFA tubing (1 mL volume). Using a flow rate of 500 µL/min in the feed containing the bromoacetyl bromide solution – corresponding to 1.0 equiv of the bromide with respect to the starting vinyl azide – both oxazoles **6a** and **6b** were obtained in full conversion, with 81% and 59% purity (HPLC). Unfortunately, the oxazoles could not be precipitated as hydrobromide salts even after cooling at −20 °C and adding petroleum ether as co-solvent. The work-up consisted in extraction with aqueous NaHCO_3_, evaporation of the organic phase, and purification of the residue by column chromatography. Relatively poor isolated yields (42% and 35% for compounds **6a** and **6b**, respectively) were achieved due to decomposition of the products during isolation. The decomposition of the 2-(bromomethyl)oxazoles inside the column was apparent, both when silica or neutral alumina were used as stationary phase.

**Figure 4:**
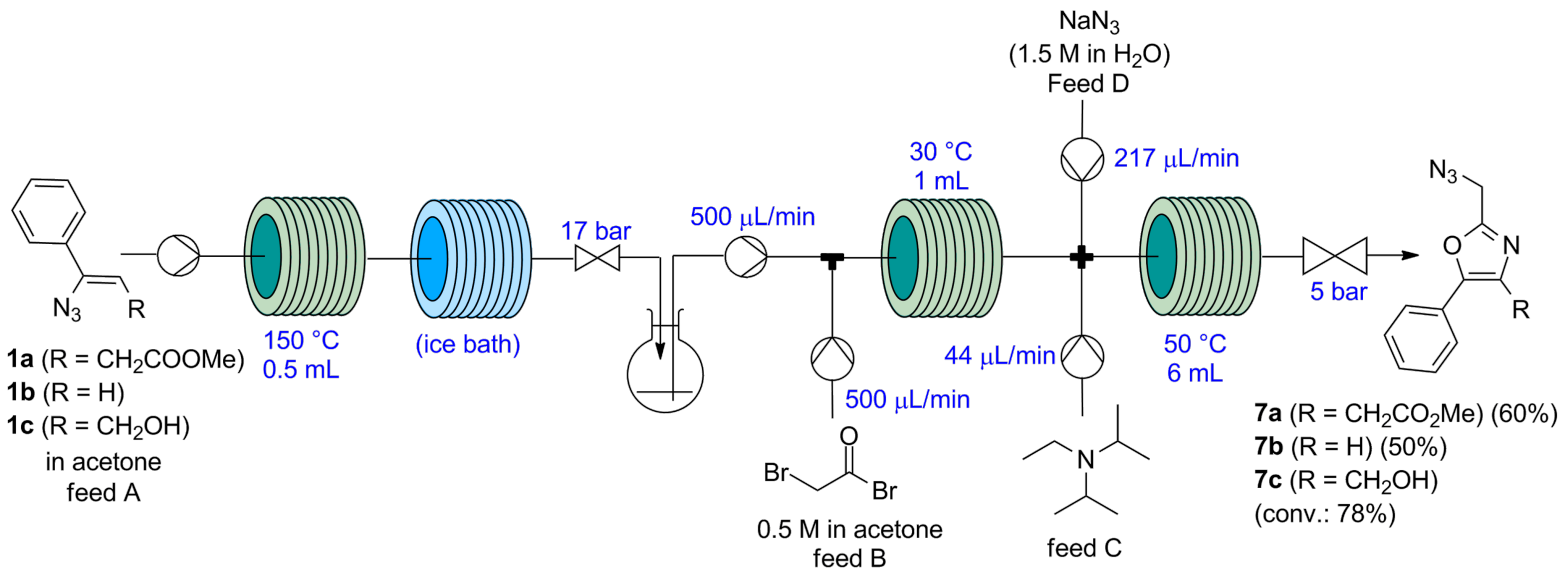
Continuous-flow three-step sequential synthesis of 2-(azidomethyl)oxazoles **7a**–**c** from vinyl azides **1a**–**c**. Yields refer to isolated yields.

Decomposition of 2-(bromomethyl)oxazoles **6** was successfully avoided by further integrating into the continuous-flow reactor the final nucleophilic halide displacement step with NaN_3_. The resulting 2-(azidomethyl)oxazole derivatives **7** presented higher stability and could be isolated without decomposition. Thus, two additional reagents streams were added to the flow setup (Figure 4) containing an aqueous solution of NaN_3_ (1.5 M) and DIPEA, respectively. The three streams were mixed in a cross mixer before entering a coil reactor at 50 °C (PFA tubing, 6 mL). While the vinyl azide thermolysis reactor zone was pressurized at 250 psi (17 bar), for this reactor zone 75 psi (5 bar) sufficed. Using this continuous-flow setup, azido oxazoles **7a** and **7b** were prepared from vinyl azides **1a** and **1b** in a three-step sequence (azirine was not isolated, the solution of the generated azirine was directly employed in the reaction described above). After reaction, 2-(azidomethyl)oxazoles **7a** and **7b** were purified by column chromatography, giving a three-step overall yield of 60% and 50%, respectively.

The vinyl azide **1c** was also subjected to the conditions described above. However, the reaction could not be completed due to solid generation in the second reactor zone (likely the hydrobromide salt of the oxazole). The reactor clogging could not be avoided either by sonication of the tubing or increasing the temperature to 50 °C. Thus, the reaction was performed employing a 0.25 M solution of substrate **1c**. Under diluted conditions the reaction mixture remained fully homogeneous but no full conversion from bromo oxazole **6c** to azido oxazole **7c** was achieved (78%), which prevented the formation of the final product in a pure form.

## Conclusion

We have developed a continuous-flow protocol for the preparation of 2-(azidomethyl)oxazoles. The procedure consists of a three-step sequential synthesis combining an initial thermolysis of the starting vinyl azide to form an azirine intermediate, followed by reaction with bromoacetyl bromide to generate the oxazole moiety, and a final nucleophilic halide displacement with NaN_3_ to give the desired product. After optimization of all individual steps in batch and continuous-flow mode, the complete sequence has been integrated in a single continuous-flow reactor, in which the vinyl azide is fed as substrate and the final 2-(azidomethyl)oxazole is formed and collected from the reactor output. The process avoids the isolation and handling of the unstable 2-(bromomethyl)oxazole intermediates, thus circumventing decomposition problems. The continuous reactor has been tested for three different vinyl azide substrates. Good results were obtained for compounds **7a** and **7b**, while for **7c** dilution was necessary to avoid clogging of the reactor.

## Supporting Information

File 1 Experimental procedures and copies of the NMR spectra for all isolated compounds.
