# Supplementary material for: Continuous multistep synthesis of 2-(azidomethyl)oxazoles
Source: Beilstein J Org Chem. 2018 Feb 23;14:506–14. doi: 10.3762/bjoc.14.36 (PMC5827817; doi:10.3762/bjoc.14.36)
Supplement: File 1 — Experimental procedures and copies of the NMR spectra for all isolated compounds. [file Beilstein_J_Org_Chem-14-506-s001.pdf]

Supporting Information

for

Continuous multistep synthesis of 2-(azidomethyl)oxazoles

Thaís A. Rossa[1,2], Nícolas S. Suveges[3], Marcus M. Sá[2], David Cantillo[1,4]* and C. Oliver Kappe[1,4]*

Address: [1]Institute of Chemistry, University of Graz, NAWI Graz, Heinrichstrasse 28, 8010 Graz, Austria, [2]Departamento de Química, Universidade Federal de Santa Catarina, Florianópolis, SC, Brazil 88040-900, [3]Chemistry Institute, Federal University of Rio de Janeiro, Rio de Janeiro, RJ, Brazil 22941-909, and [4]Research Center Pharmaceutical Engineering GmbH (RCPE), Inffeldgasse 13, 8010 Graz, Austria

Email: David Cantillo - david.cantillo@rcpe.at; C. Oliver Kappe - oliver.kappe@uni-graz.at

*Corresponding author

Experimental procedures and copies of the NMR spectra for all isolated compounds

Contents

Experimental procedures S2
Copies of NMR spectra for all isolated compounds S9

Experimental procedures

General procedures. ^{1}H NMR spectra were recorded with a Bruker 300 MHz spectrometer. ^{13}C NMR spectra were recorded with the same instrument at 75 MHz. Chemical shifts (δ) are expressed in ppm downfield from TMS as internal standard. The letters s, d, t, q, and m are used to indicate singlet, doublet, triplet, quadruplet, and multiplet. Analytical HPLC–UV–vis (Shimadzu LC20) analysis was carried out with a C18 reversed-phase (RP) analytical column (150 × 4.6 mm, particle size 5 µm) at 37 °C by using mobile phases A [water/acetonitrile 90:10 (v/v) + 0.1 % TFA] and B (MeCN + 0.1 % TFA) at a flow rate of 0.6 mL/min (the following gradient was applied: linear increase from 5% B to 100% B in 15 min, total analysis time: 25 min). Column chromatography was performed using silica gel (60 mesh) or alumina (neutral, 60 mesh) as the stationary phase and petroleum ether/ethyl acetate as the eluent. Vinyl azides **1a**–**c** were prepared according to known literature protocols [S1-S3].
CAUTION! Organic azides are potentially explosive and should be handled with care, although we experienced no problems in handling solutions of sodium azide or organic azides, which were stored under refrigeration.

Sealed vessel batch thermolysis of vinyl azide 1a. Into an HPLC vial equipped with a magnetic stirring bar were placed 0.5 mL of a solution of vinyl azide **1a** in dry acetone (0.5 M solution prepared in a volumetric flask). The vial was sealed by a snap-cap and heated to the desired temperature using a dry bath aluminum heating block (Table 1). After 1 min, the vial was removed from the heating block and immediately cooled in an ice bath. The solution of the crude product **2a** was immediately analyzed by HPLC.

Sealed vessel batch synthesis of bromo oxazole 6a from azirine 2a. Into an HPLC vial equipped with a magnetic stirring bar were placed 0.5 mL of a solution of azirine **2a** in dry acetone (0.5 M). The vial was sealed by a snap-cap with septum and purged with argon. Into the closed flask under stirring 0.5 mL of a freshly prepared 0.5 M solution of bromoacetyl bromide in dry acetone were injected. The mixture was stirred at room temperature for 1 min and afterwards was immediately analyzed by LC–MS or quenched in $NaHCO_3$ aqueous solution (1.0 M). The aqueous solution was extracted with EtOAc and the organic extract was washed with H_2O, dried with anhydrous Na_2SO_4 and concentrated under reduced pressure.

Sealed vessel batch synthesis of azido oxazole 7a from azirine 2a. Into an HPLC vial equipped with a magnetic stirring bar were placed 0.4 mL of a solution of azirine **2a** in dry acetone (0.5 M). The vial was sealed by a snap-cap with septum and purged with argon. Into the closed flask under stirring 0.4 mL of a freshly prepared 0.5 M solution of bromoacetyl bromide in dry acetone were injected. The mixture was stirred at room temperature for 1 min. Afterwards, the vial was opened and 35 µL of DIPEA (1.0 equiv) and 173 µL of aqueous NaN_3 (1.5 M, 1.3 equiv) were added, the vial closed and the reaction mixture stirred at 50 °C for 5 min. The crude mixture was immediately analyzed by LC–MS.

Synthesis of azido oxazole 7c from vinyl azide 1c in batch. In a microwave tube were placed 1.2 mL of a solution of vinyl azide **1c** in acetone (0.5 M). The sealed tube was heated under microwave radiation (150 °C) for 1 min. After cooling the mixture was diluted with 1.2 mL of acetone and bromoacetyl bromide (1.0 equiv) was added to the stirred solution. After 1 min, DIPEA (1.0 equiv) and 520 µL of aqueous NaN_3 (1.5 M, 1.3 equiv) were added, the tube closed and the reaction mixture heated

under microwave radiation (50 °C) for 5 min. Then, the mixture was diluted with EtOAc and the organic phase was washed with H_2O, dried with anhydrous Na_2SO_4, and concentrated under reduced pressure. The crude product was purified by column chromatography (hexane/EtOAc 7:3) to give the desired vinyl azide **7c**.

Continuous-flow synthesis of azirines 2 from vinyl azides 1. The flow experiments were performed using the continuous-flow setup depicted in Table 4. The reactor was preheated using convective heating with a silicon bath at 150 °C and the system was washed with dry acetone. A 0.5 M solution of vinyl azide **1a**–**c** in acetone was pumped into the reactor at the appropriate flow rate (Table 4) and immediately cooled in a second coil maintained in an ice bath. After steady state conditions were achieved, 5 mL of the crude product (2.5 mmol) were collected from the reactor output into a closed flask under argon atmosphere and used in the next step reaction without any purification. In addition, the solution collected from the reactor output was immediately analyzed by LC–MS.

Continuous-flow synthesis of bromo oxazoles 6 from azirines 2. The flow experiments were performed using the continuous-flow setup depicted in Figure 3. The reactor temperature was stabilized at 30 °C using a water bath and the system was washed with dry acetone. In the feed A was pumped 0.5 M solution of azirine **2a** or **2c** (500 µL/min) and in the feed B was pumped a freshly prepared 0.5 M solution of bromoacetyl bromide in dry acetone (500 µL/min), both were prepared using dry acetone. The feeds were mixed in a Y-shaped mixer and the combined solution went through the coil reactor. Under steady state conditions, 8 mL of the reaction mixture were collected from the reactor output into a graduated test tube. The solution was

diluted with EtOAc and the organic extract was washed with 1.0 M $NaHCO_3$ (aq), H_2O and brine, dried with anhydrous Na_2SO_4, and concentrated under reduced pressure.

Continuous-flow synthesis of azido oxazoles 7 from azirines 2. The flow experiments were performed using the continuous-flow setup depicted in Figure 4. The temperature of the first and the second reactor was stabilized at 30 °C and 50 °C, respectively. Feeds A, B and C were rinsed with dry acetone while feed D carried water. Feed A contained an 0.5 M solution of azirine **2a** or **2b** in dry acetone. Feed B consisted in a 0.5 M solution of bromoacetyl bromide freshly prepared in dry acetone. Feed C consisted in pure *N,N*-diisopropylethylamine (DIPEA). Feed D contained the aqueous solution of NaN_3 (1.5 M). The following flow rates were used: Feed A and B: 500 µL/min, feed C: 44 µL/min and feed D: 217 µL/min. The solutions from feeds A and B were mixed in a Y-shaped mixer and the combined mixture went through the coil reactor. The reactor output was combined with feeds C and D in a second mixer and the resulting solution went through another coil reactor. Six mL of the reaction mixture were collected from the reactor output into a graduated test tube. In order to avoid concentration variations, the initial and final fraction were discarded. The solution was diluted with EtOAc, the organic phase was washed with H_2O and brine, dried with anhydrous Na_2SO_4, and concentrated under reduced pressure. Purification was performed by automated flash chromatography, eluting with a gradient PE/EtOAc from 7:3 to 5:5.

Methyl 3-phenyl-2*H*-azirine-2-acetate (2a) [S1]**.** ^{1}H NMR (300 MHz, $CDCl_3$) δ 7.95 – 7.92 (m, 2H), 7.64 – 7.50 (m, 3H), 3.70 (s, 3H), 2.87 (dd, *J* = 16.3, 4.7 Hz, 1H), 2.50 (dd, *J* = 5.8, 4.7 Hz, 1H), 2.36 (dd, *J* = 16.3, 5.8 Hz, 1H).

3-Phenyl-2*H*-azirine (2b) [S4]. ^{1}H NMR (500 MHz, $CDCl_3$): δ 7.90 (dd, *J* = 8.0, 1.6 Hz, 2H), 7.59-7.54 (m, 3H), 1.79 (s, 2H).

3-Phenyl-2*H*-azirine-2-methanol (2c) [S2]. ^{1}H NMR (500 MHz, $CDCl_3$): δ 7.91 – 7.84 (m, 2H), 7.60 – 7.52 (m, 3H), 3.97 (dd, *J* = 12.4, 3.0 Hz, 1H), 3.69 (dd, *J* = 12.4, 5.1 Hz, 1H), 2.47 (dd, *J* = 5.1, 3.0 Hz, 1H), 2.26 (br s, 1H).

Methyl 2-(bromomethyl)-5-phenyloxazol-4-acetate (6a). Yellow solid, mp 76.3-78.1 °C; ^{1}H NMR (300 MHz, $CDCl_3$): δ 7.63 – 7.59 (m, 2H), 7.49 – 7.34 (m, 3H), 4.49 (s, 2H), 3.77 (s, 2H), 3.75 (s, 3H). ^{13}C NMR (75 MHz, $CDCl_3$): δ 170.4 (C), 157.6 (C), 149.2 (C), 129.6 (C), 129.1 (3 × CH), 127.7 (C), 126.2 (2 × CH), 52.6 (CH_3), 33.3 (CH_2), 20.6 (CH_2); HRMS (ESI+): calcd. for $C_{13}H_{13}BrNO_3^+$ $[M+H]^+$ 310.0073; found 310.0075.

2-(Bromomethyl)-5-phenyloxazole (6b) [S5]. Yellow solid, mp 87.0-88.3 °C; ^{1}H NMR (500 MHz, $CDCl_3$): δ 7.65 (d, *J* = 7.5 Hz, 2H), 7.43 (t, *J* = 7.5 Hz, 2H), 7.35 (t, *J* = 7.5 Hz, 1H), 7.32 (s, 1H), 4.53 (s, 2H); ^{13}C NMR (126 MHz, $CDCl_3$): δ 158.7 (C), 152.9 (C), 129.1 (2 × CH), 129.0 (CH), 127.6 (C), 124.6 (2 × CH), 122.9 (CH), 20.7 (CH_2); HRMS (ESI+): calcd. for $C_{10}H_9BrNO^+$ $[M+H]^+$ 237.9862; found 237.9863.

2-(Bromomethyl)-5-phenyloxazol-4-methanol (6c). Yellow solid, mp 65-67 °C.
^{1}H NMR (200 MHz, $CDCl_3$): δ 7.67 (dd, *J* = 8.1, 1.5 Hz, 2H), 7.50 – 7.38 (m, 3H), 4.75 (s, 2H), 4.48 (s, 2H), 3.77 (br s, 1H). ^{13}C NMR (50 MHz, $CDCl_3$): δ 157.7 (C), 149.0 (C), 135.7 (C), 129.2 (CH), 129.1 (2 × CH), 127.5 (C), 126.4 (2 × CH), 56.7 (CH_2), 20.2 (CH_2). HRMS (ESI+): calcd. for $C_{11}H_{10}BrNO_2Na^+$ $[M+Na]^+$ 289.9787; found 289.9786.

Methyl 2-(azidomethyl)-5-phenyloxazol-4-acetate (7a). 194 mg (60% overall yield after three steps from vinyl azide **1a**); yellow oil. ^{1}H NMR (500 MHz, $CDCl_3$): δ 7.60 (d, *J* = 7.2 Hz, 2H), 7.45 (apt, *J* = 7.6 Hz, 2H), 7.38 (t, *J* = 8.0 Hz, 1H), 4.46 (s, 2H), 3.78 (s, 2H), 3.74 (s, 3H); ^{13}C NMR (126 MHz, $CDCl_3$): δ 170.4 (C), 157.1 (C), 149.0 (C), 129.13 (C), 129.07 (2 × CH), 129.0 (CH), 127.7 (C), 126.2 (2 × CH), 52.5 (CH_3), 46.8 (CH_2), 33.3 (CH_2); HRMS (ESI+): calcd. for C13H13N4O3$^+$ $[M+H]^+$ 273.0982; found 273.0984.

2-(Azidomethyl)-5-phenyloxazole (7b). 119 mg (50% overall yield after three steps from vinyl azide **1b**); colorless oil. ^{1}H NMR (500 MHz, $CDCl_3$): 7.64 (d, *J* = 7.5 Hz, 2H), 7.43 (t, *J* = 7.5 Hz, 1H), 7.35 (t, *J* = 7.5 Hz, 1H), 7.32 (s, 1H), 4.48 (s, 2H). ^{13}C NMR (126 MHz, $CDCl_3$): 158.2 (C), 152.8 (C), 129.1 (2 × CH), 129.0 (CH), 127.6 (C), 124.5 (2 × CH), 122.3 (CH), 46.9 (CH_2). HRMS (ESI+): calcd. for $C_{10}H_9N_4O^+$ $[M+H]^+$ 201.0771; found 201.0772.

2-(Azidomethyl)-5-phenyloxazol-4-methanol (7c). 91 mg (66% overall yield after three steps from vinyl azide **1c** in batch process); orange oil. ^{1}H NMR (200 MHz, $CDCl_3$): δ 7.66 (dd, *J* = 8.1, 1.5 Hz, 2H), 7.50 – 7.38 (m, 3H), 4.76 (s, 2H), 4.44 (s, 2H), 3.88 (br s, 1H). ^{13}C NMR (50 MHz, $CDCl_3$): δ 157.2 (C), 148.9 (C), 135.2 (C), 129.1 (2 × CH), 127.6 (C), 126.4 (2 × CH), 56.8 (CH_2), 46.7 (CH_2). HRMS (ESI+): calcd. for $C_{11}H_{10}N_4O_2Na^+$ $[M+Na]^+$ 253.06960; found 253.06962.

References

[S1] Sá, M. C. M.; Kascheres, A. *J. Org. Chem.* **1996**, *61*, 3749-3752.

[S2] Sakai, T.; Kawabata, I.; Kishimoto, T.; Ema, T.; Utaka, M. *J. Org. Chem.* **1997**, *62*, 4906-4907.

[S3] Xiang, L.; Niu, Y.; Pang, X.; Yang, X.; Yan, R. *Chem. Commun.* **2015**, *51*, 6598-6600.

[S4] Loy, N. S. Y.; Kim, S.; Park, C.-M. *Org. Lett.* **2015**, *17*, 395-397.

[S5] Shvaika, O. P.; Klimisha, G. P. *Khim. Geterotsikl. Soedin.* **1966**, *2*, 677-681.

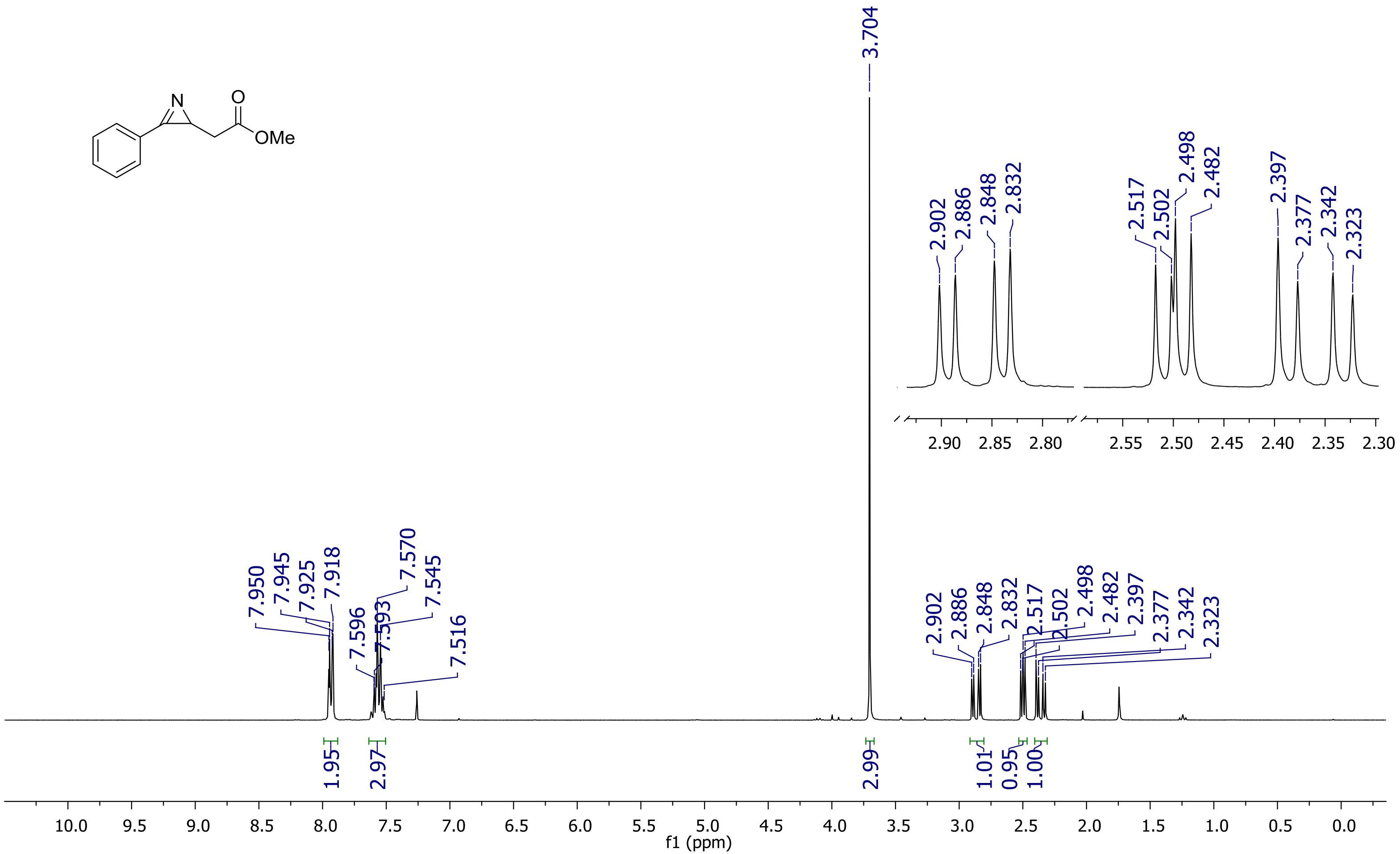

Figure S1: ^{1}H NMR (300 MHz, $CDCl_3$) of **2a**.

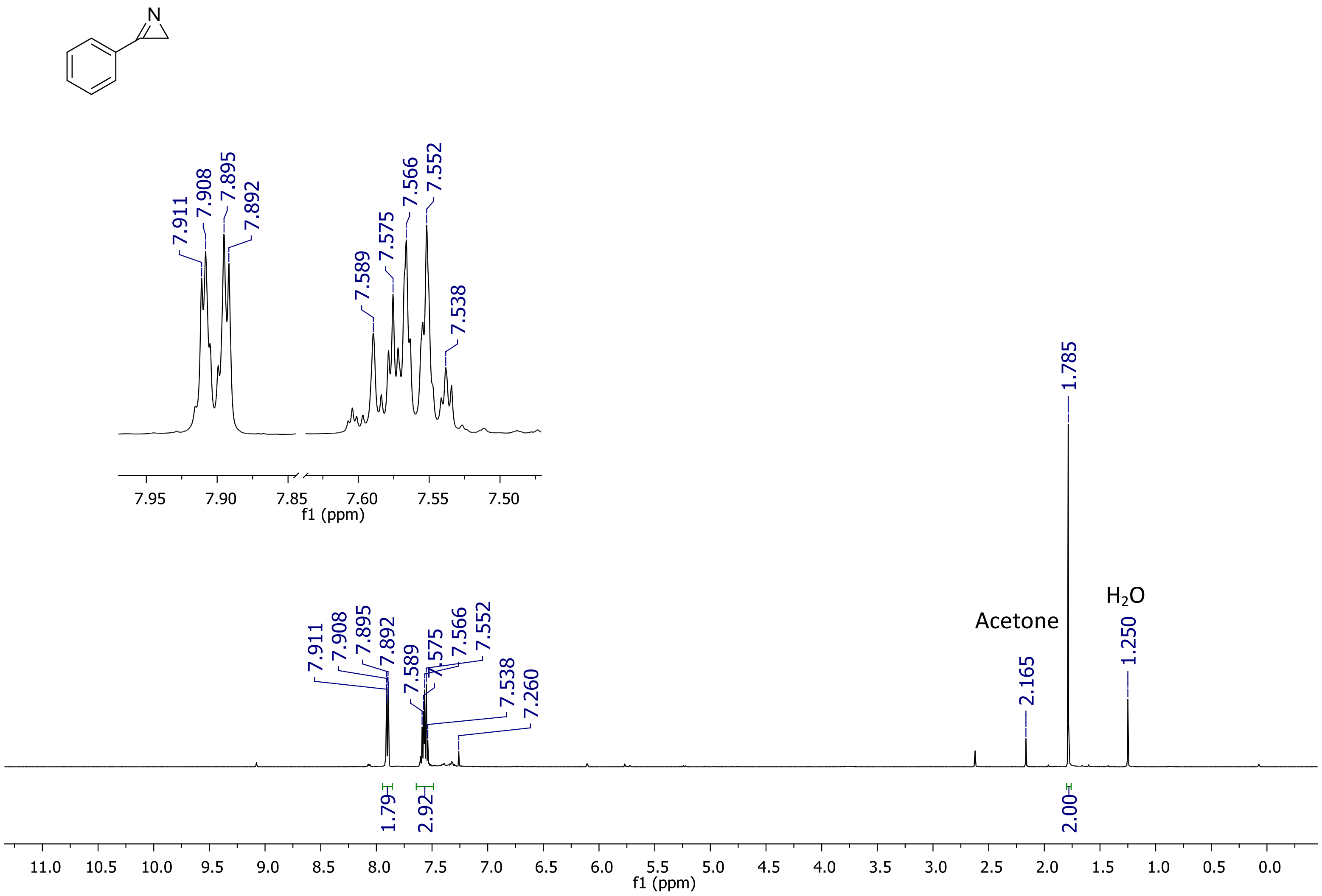

Figure S2: ^{1}H NMR (500 MHz, $CDCl_3$) of **2b**.

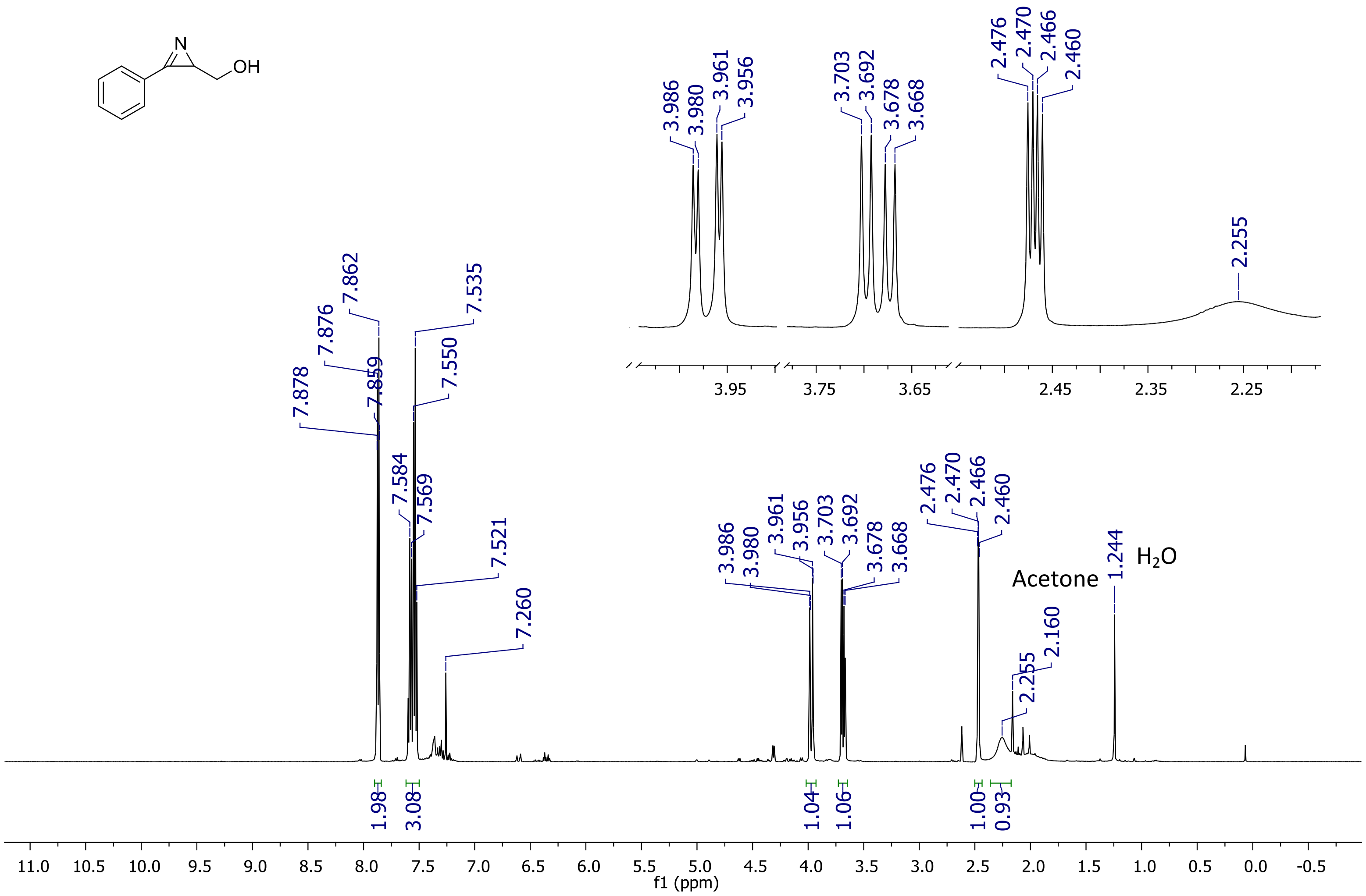

Figure S3: ^{1}H NMR (500 MHz, $CDCl_3$) of **2c**.

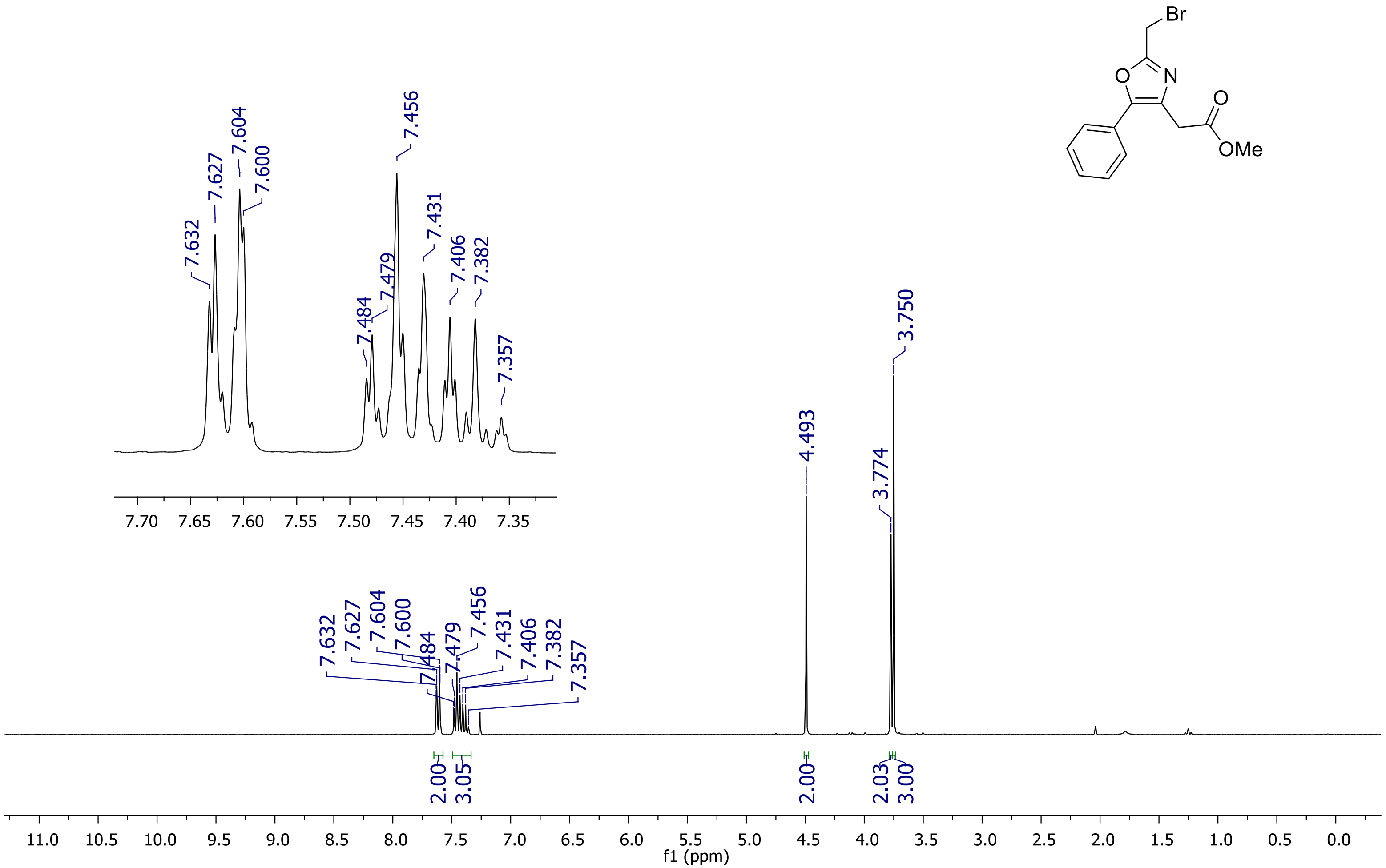

Figure S4: ^{1}H NMR (300 MHz, $CDCl_3$) of **6a**.

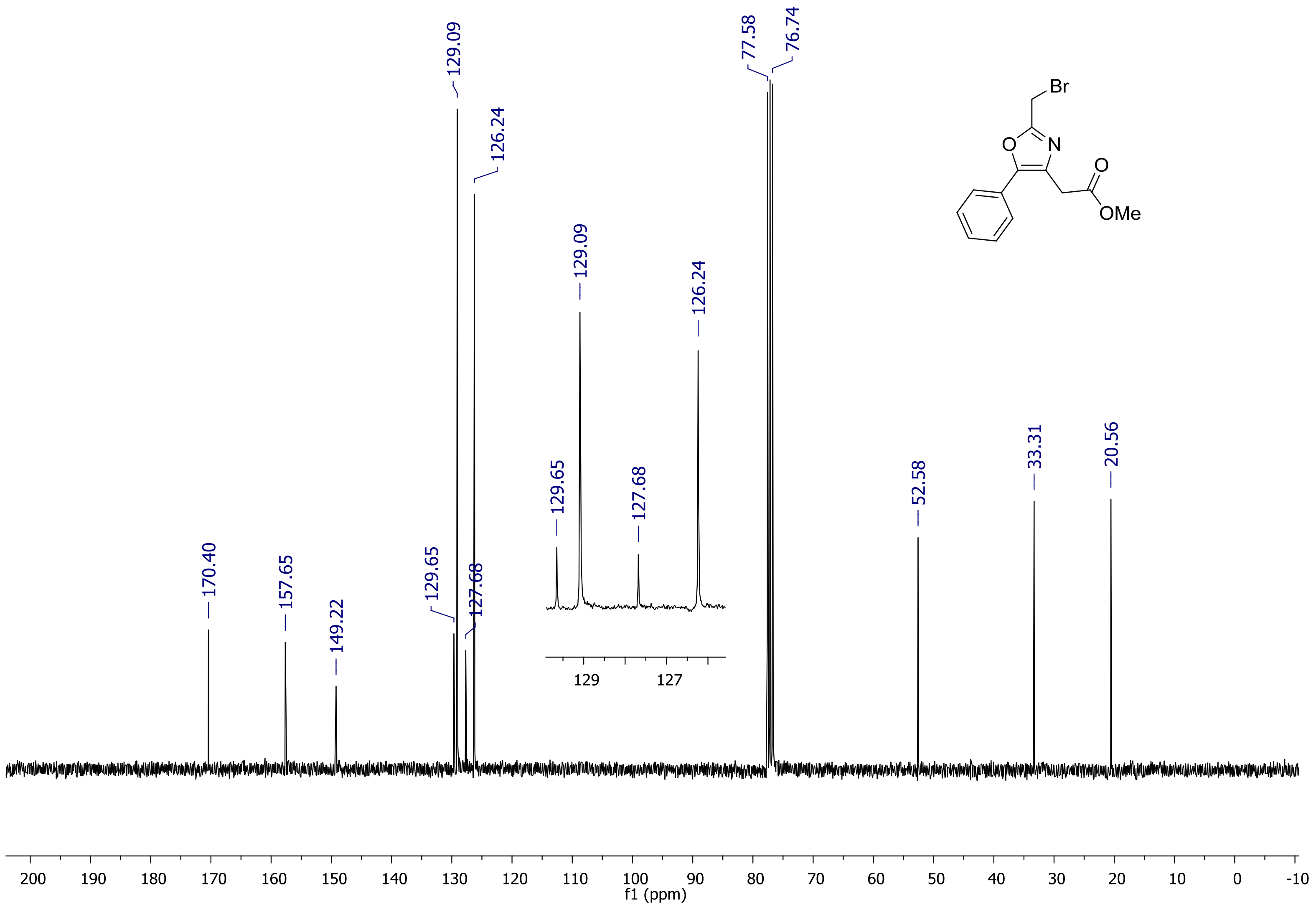

Figure S5: ^{13}C NMR (75 MHz, $CDCl_3$) of **6a**.

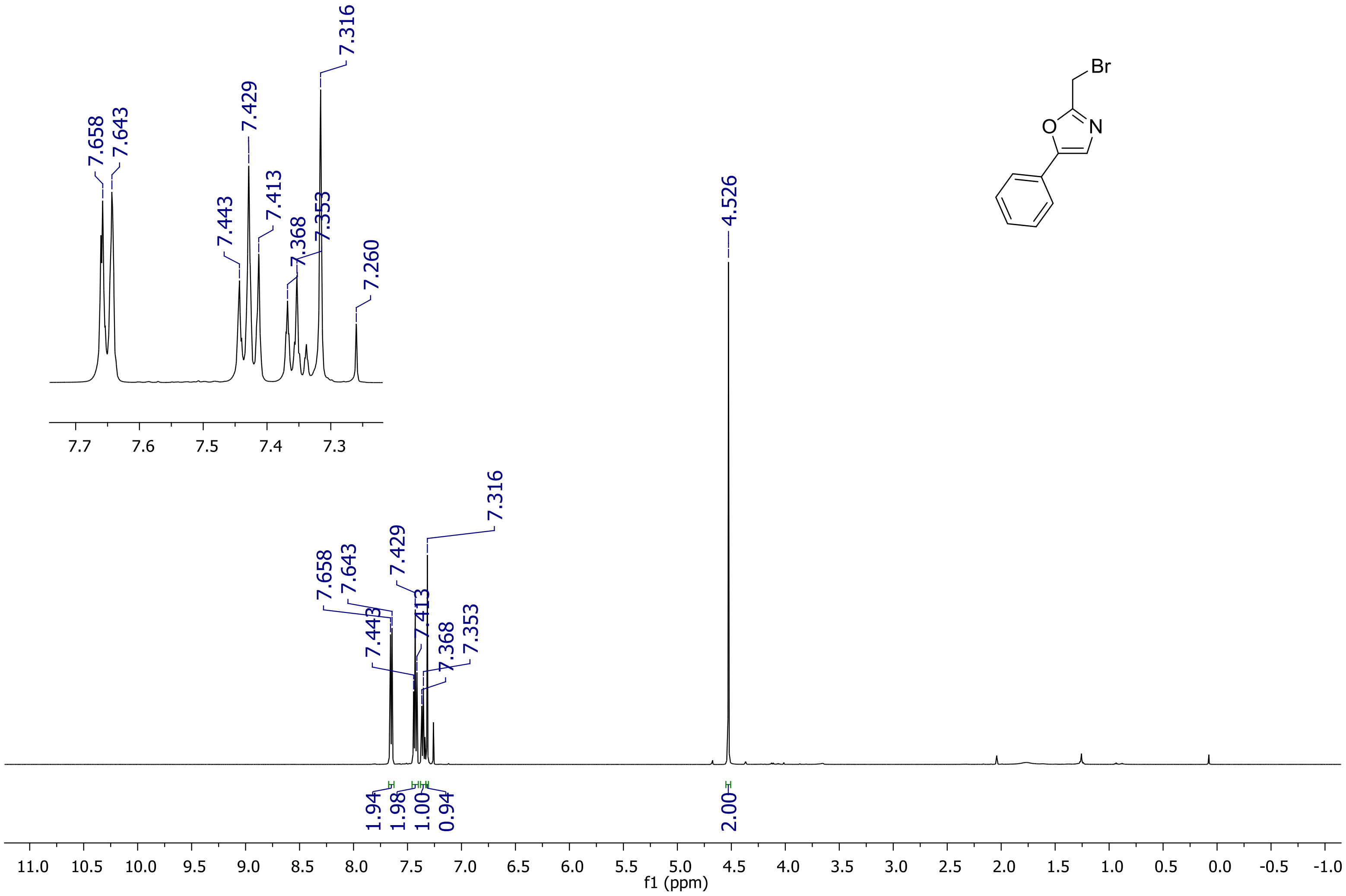

Figure S6: ^{1}H NMR (500 MHz, $CDCl_3$) of **6b**.

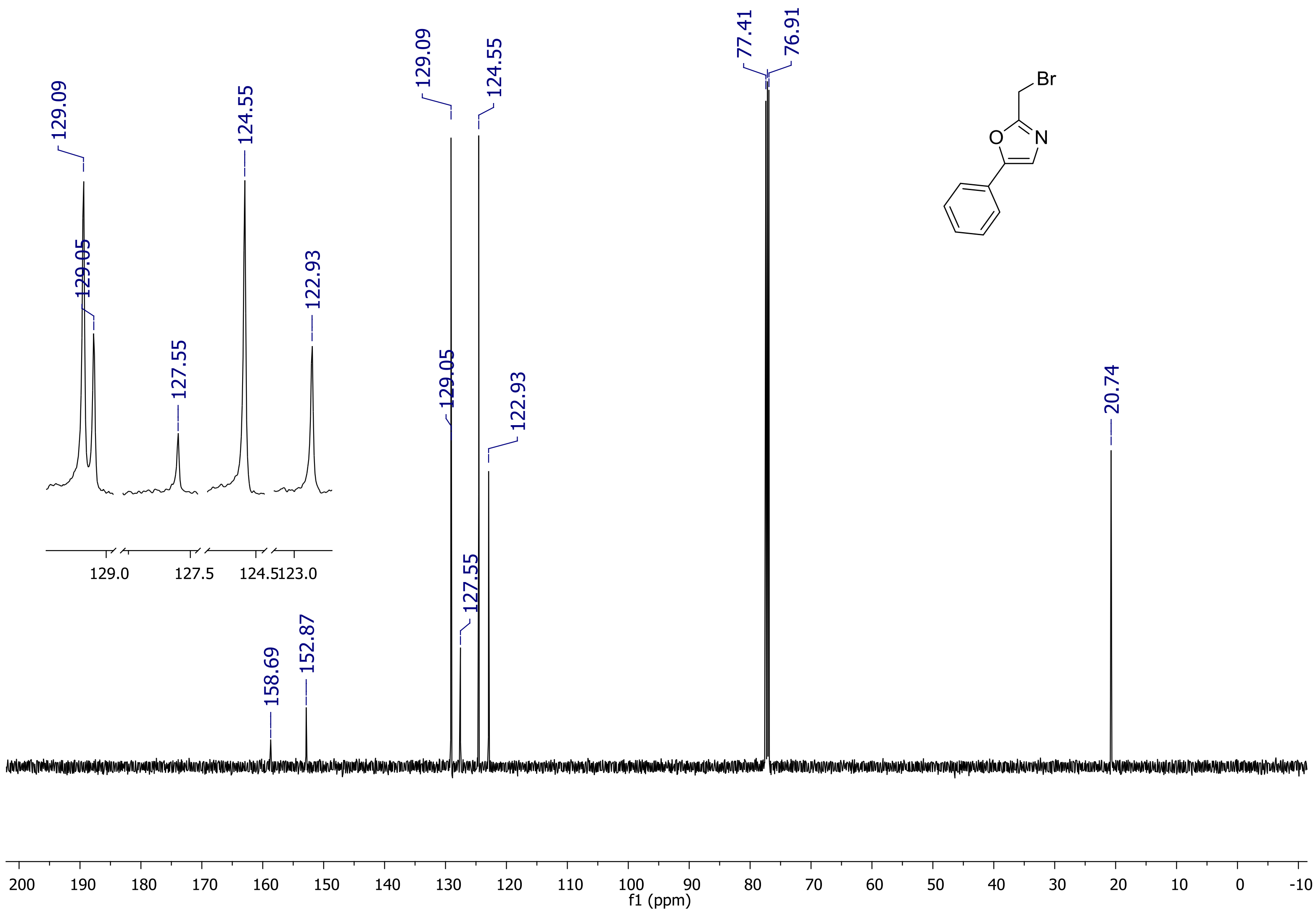

Figure S7: ^{13}C NMR (126 MHz, $CDCl_3$) of **6b**.

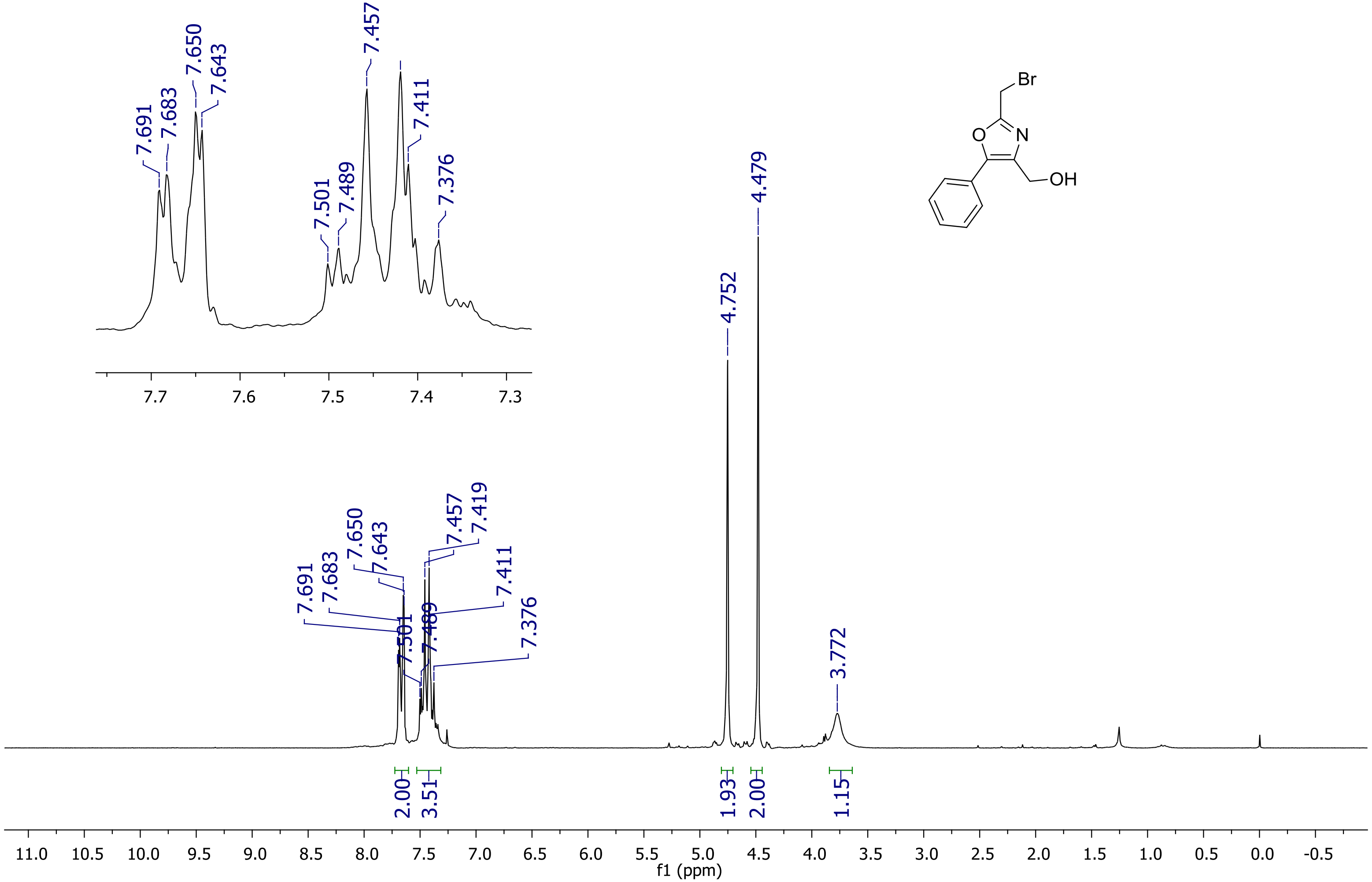

Figure S8: ^{1}H NMR (200 MHz, $CDCl_3$) of **6c**.

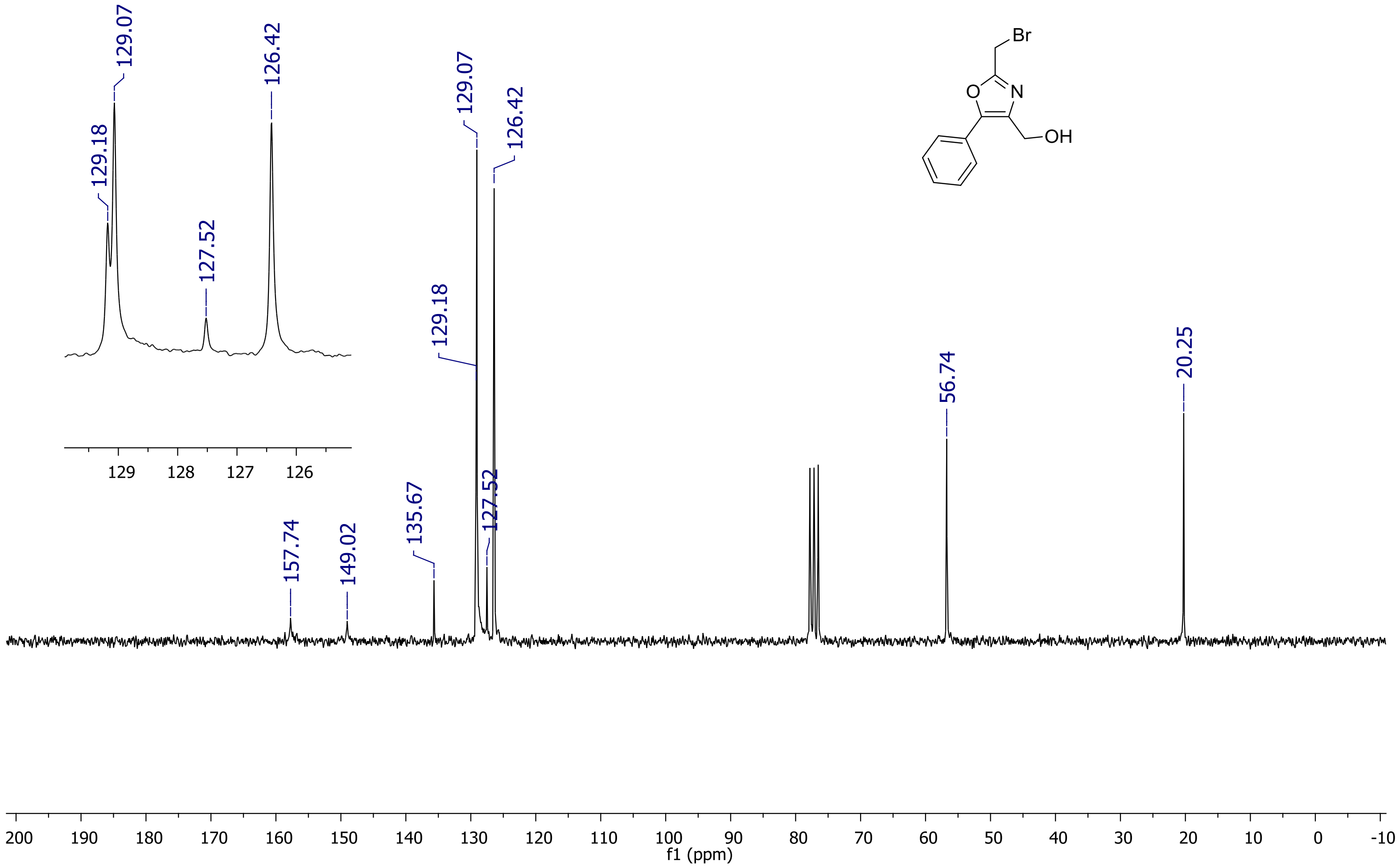

Figure S9: ^{13}C NMR (50 MHz, $CDCl_3$) of **6c**.

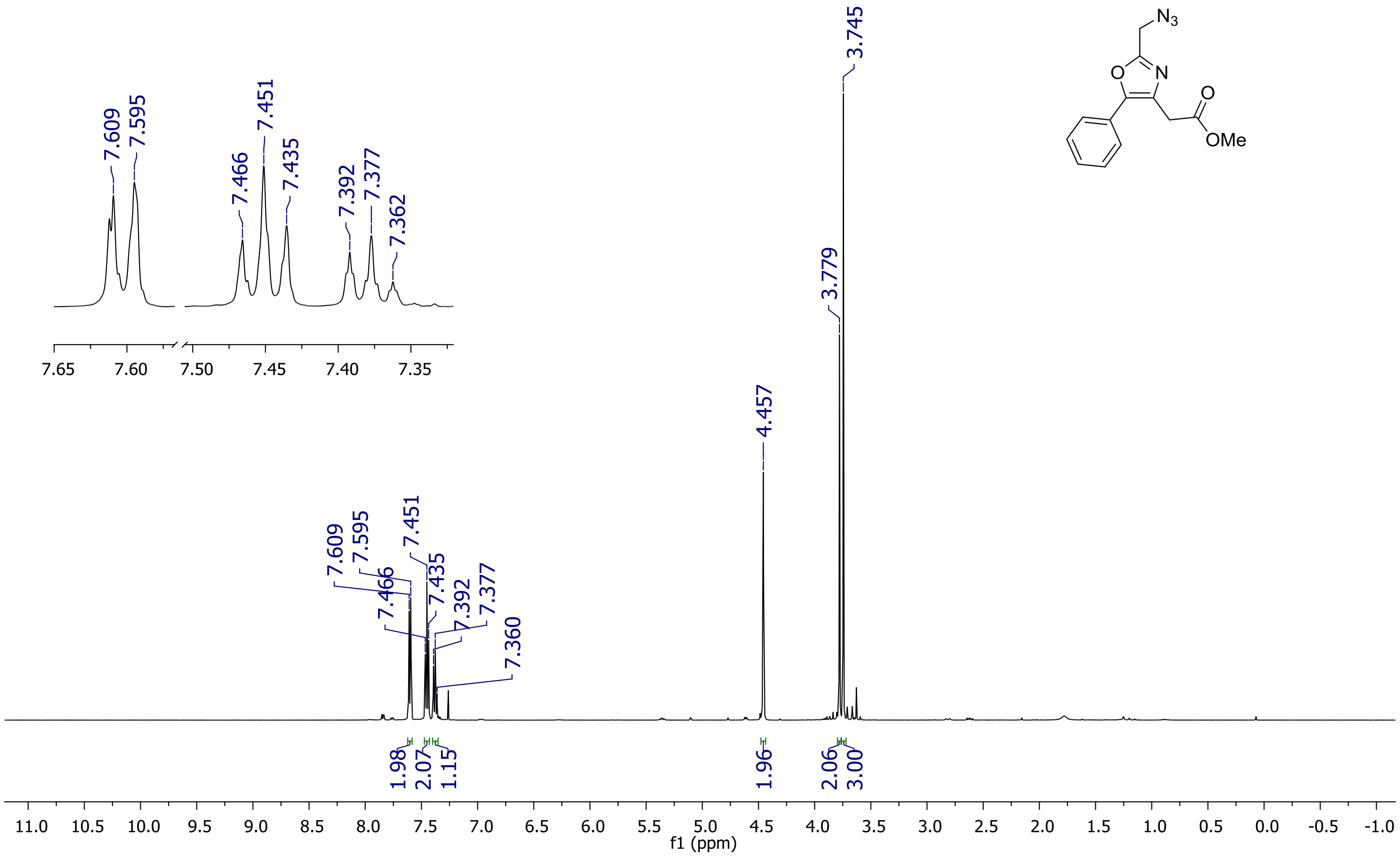

Figure S10: ^{1}H NMR (500 MHz, $CDCl_3$) of **7a**.

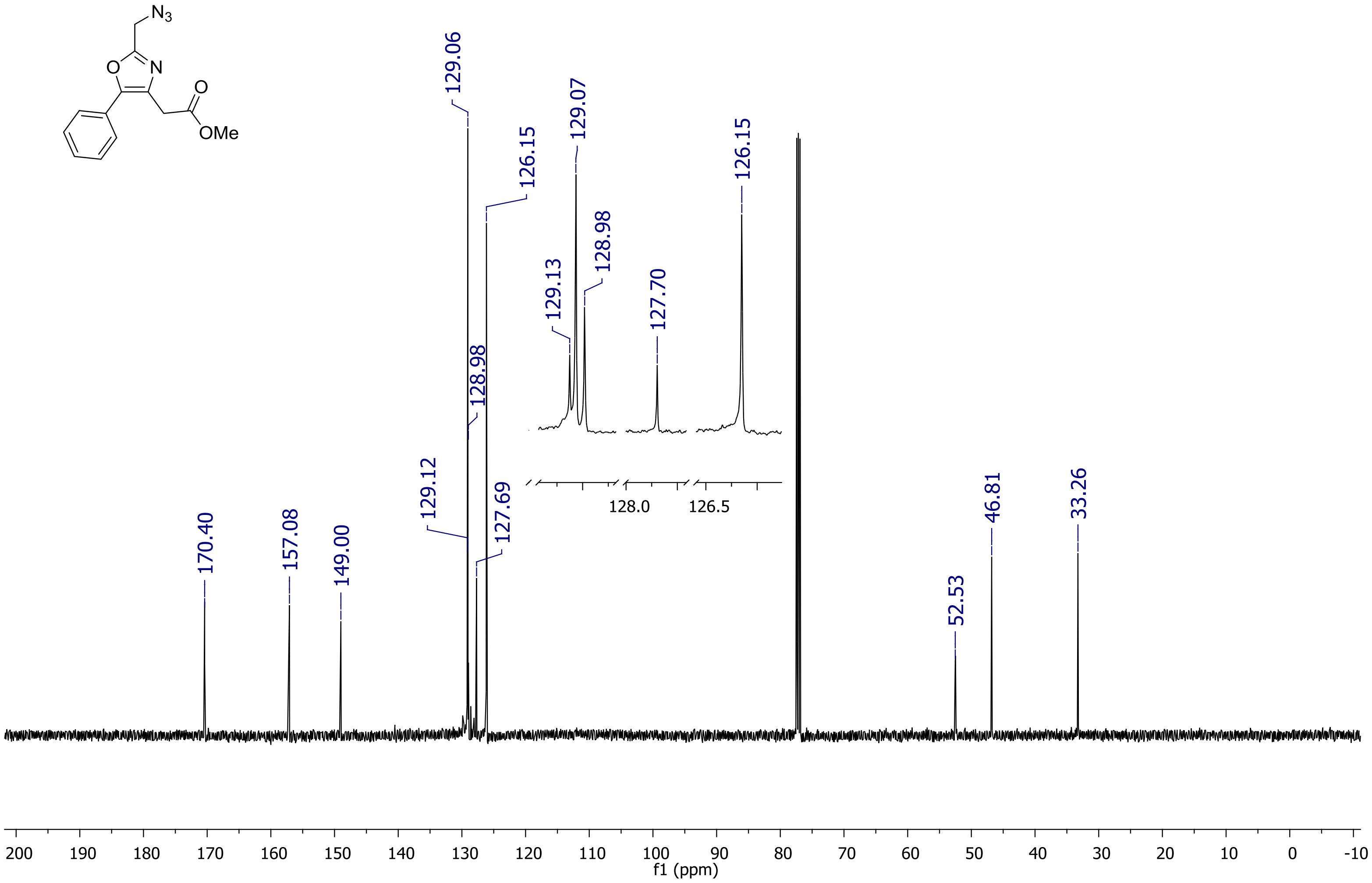

Figure S11: ^{13}C NMR (126 MHz, $CDCl_3$) of **7a**.

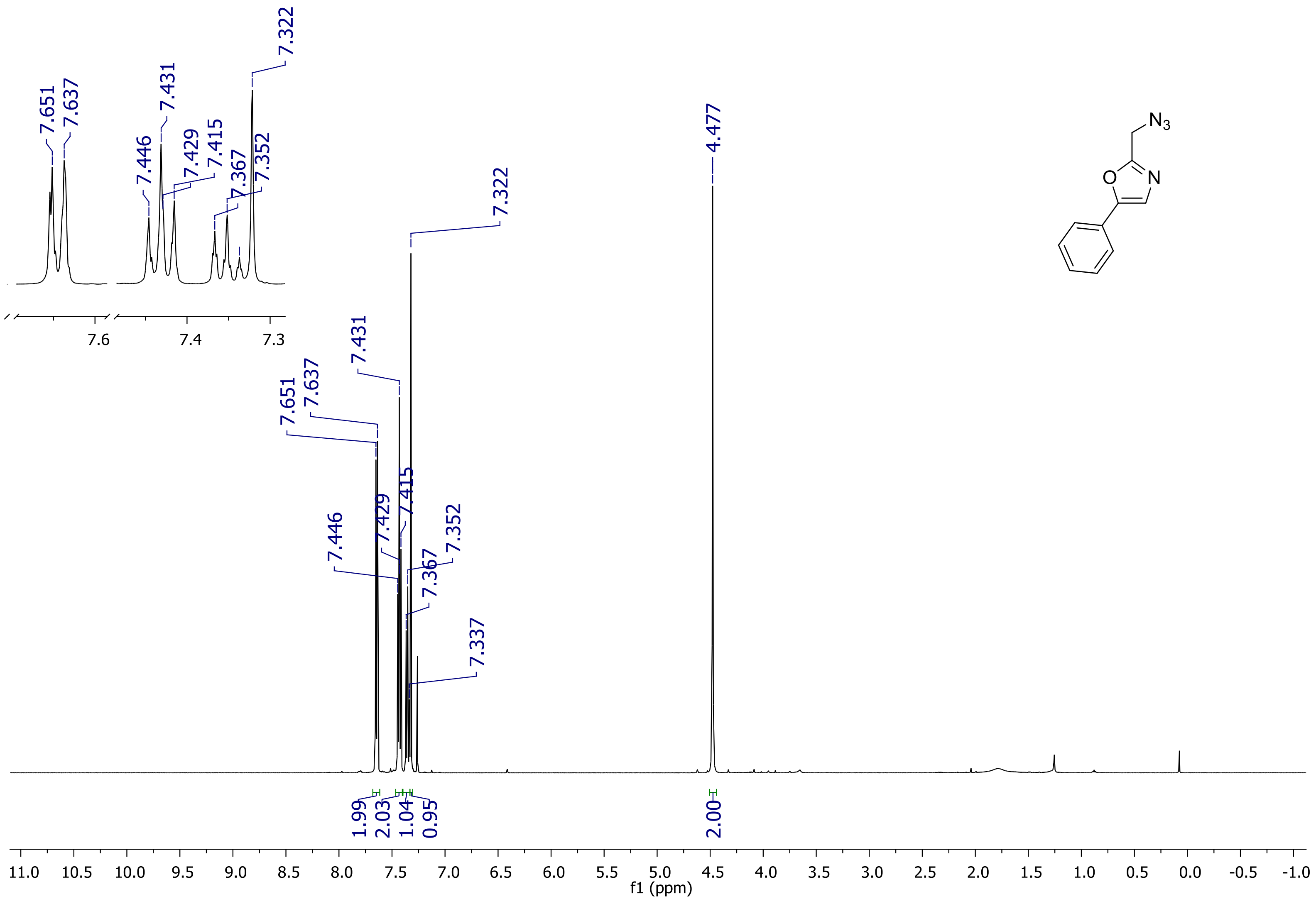

Figure S12: ^{1}H NMR (500 MHz, $CDCl_3$) of **7b**.

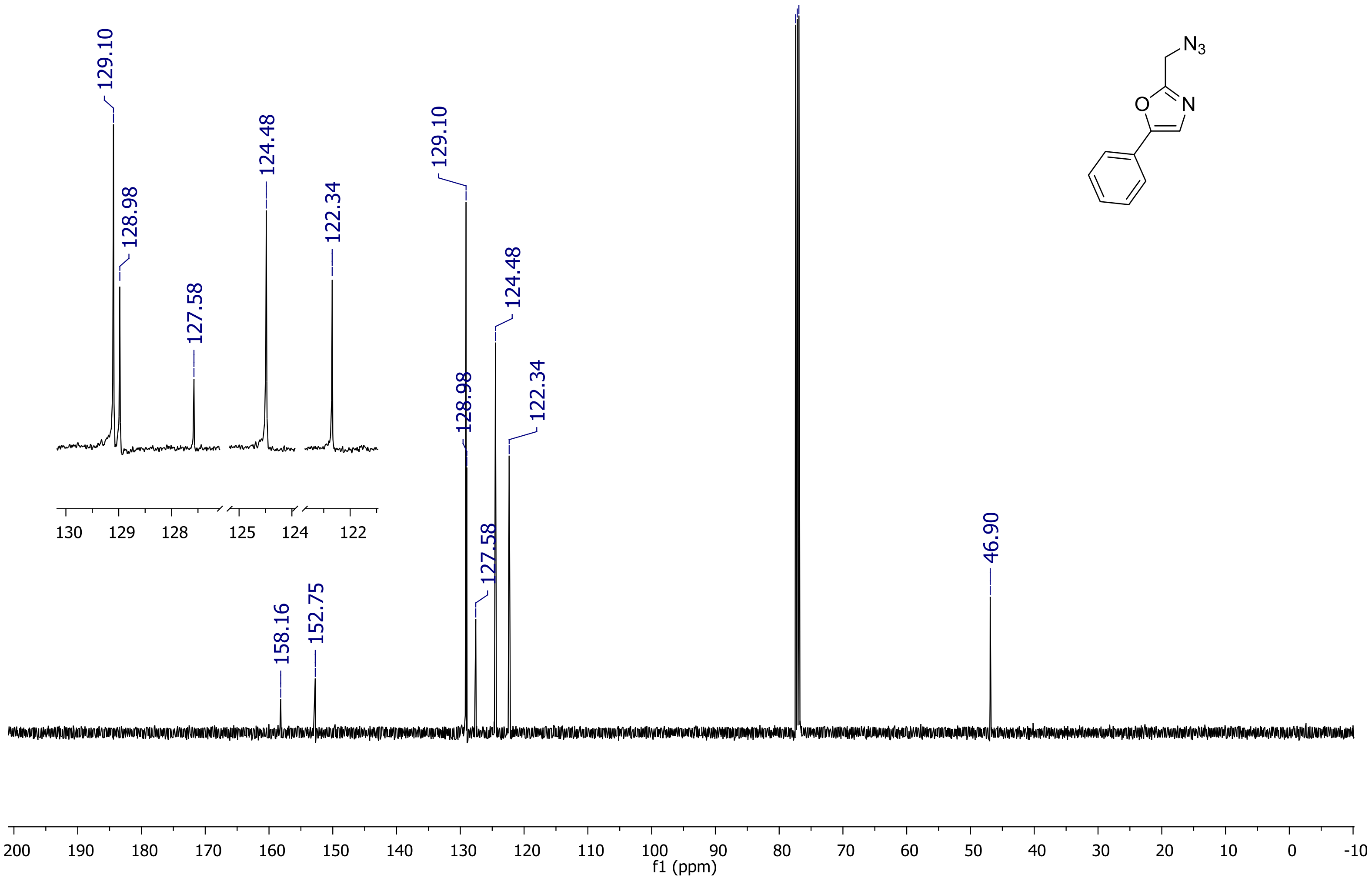

Figure S13: ^{13}C NMR (126 MHz, $CDCl_3$) of **7b**.

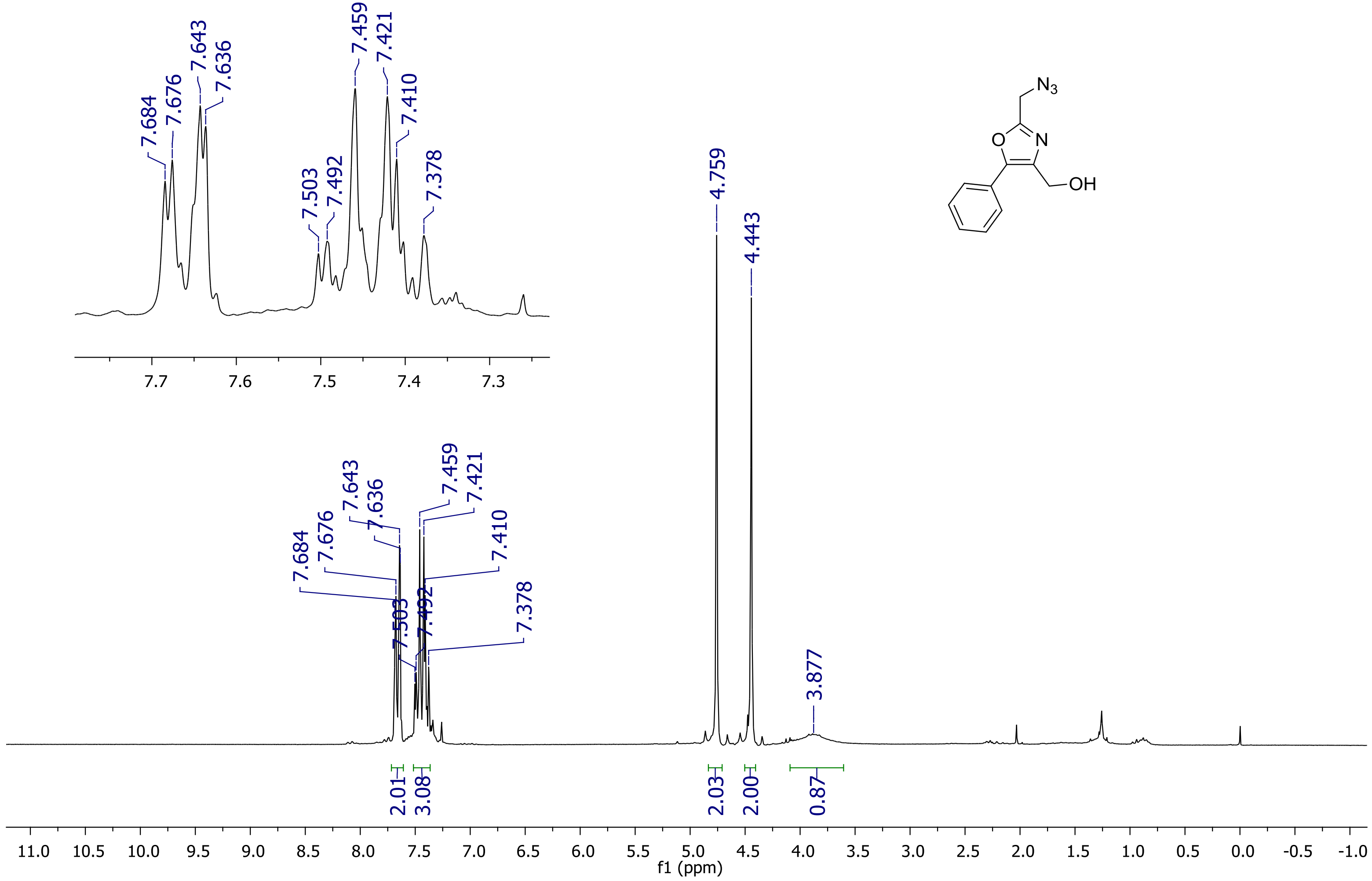

Figure S14: ^{1}H NMR (200 MHz, $CDCl_3$) of **7c**.

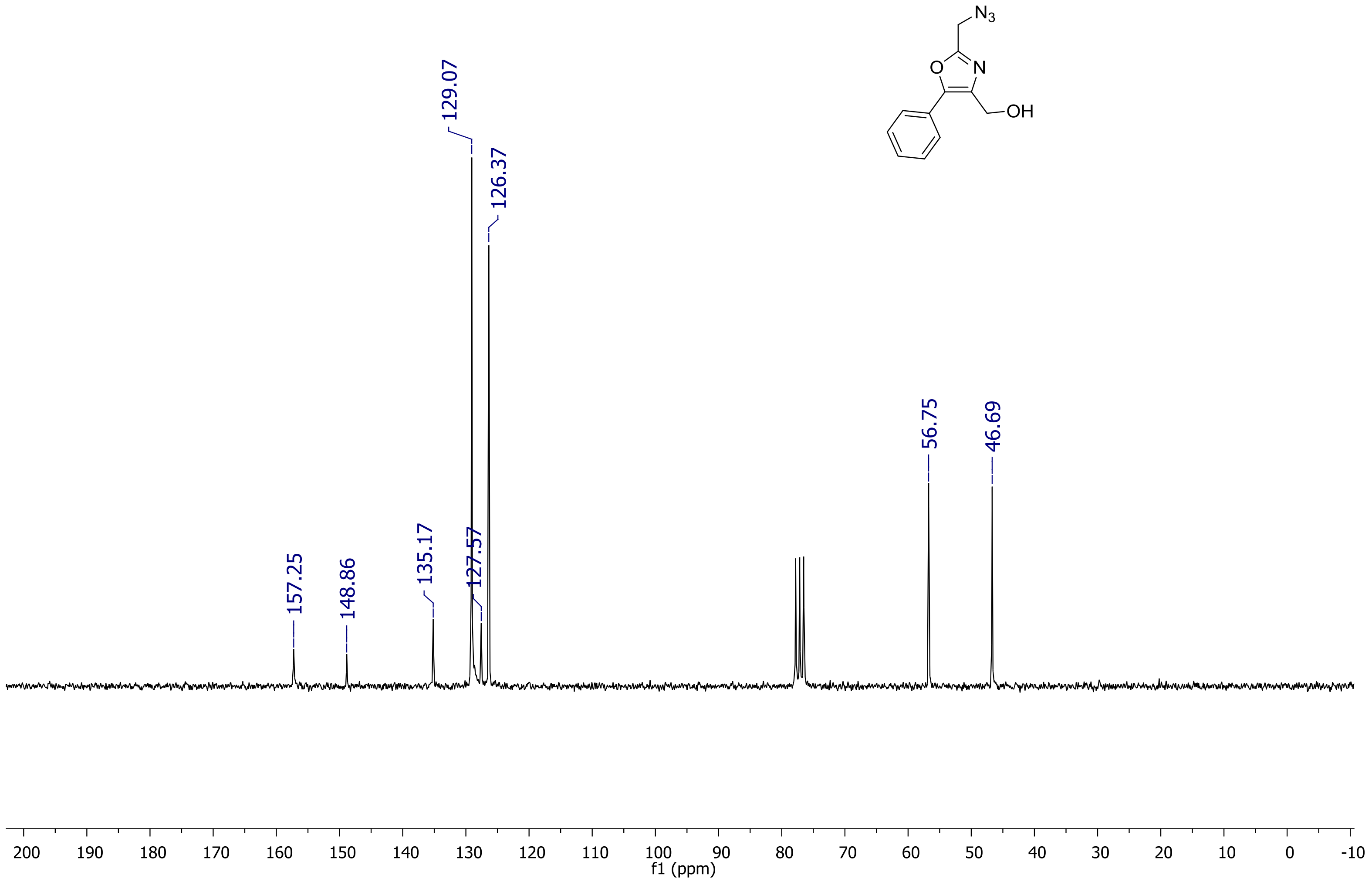

Figure S15: ^{13}C NMR (50 MHz, $CDCl_3$) of **7c**.
